# Di-μ-sulfato-κ^4^ *O*:*O*′-bis­[diaqua­(1*H*-imidazo[4,5-*f*][1,10]phenanthroline-κ^2^ *N* ^7^,*N* ^9^)cobalt(II)] dihydrate

**DOI:** 10.1107/S1600536809024295

**Published:** 2009-07-01

**Authors:** Yun Gong, Yuchao Zhou, Jinghua Li, Xiaoxia Wu, Jianbo Qin

**Affiliations:** a Department of Chemistry, College of Chemistry and Chemical Engineering, Chongqing University, Chongqing 400044, People’s Republic of China; b Department of Pharmaceutical Chemistry, College of Chemistry and Chemical Engineering, Chongqing University, Chongqing 400044, People’s Republic of China

## Abstract

In the centrosymmetric dinuclear title compound, [Co_2_(SO_4_)_2_(C_13_H_8_N_4_)_2_(H_2_O)_4_]·2H_2_O, the Co^II^ atom is coord­in­ated by two N atoms from two 1*H*-imidazo[4,5-*f*][1,10]phenanthroline ligands, two O atoms from two sulfate anions and two O atoms from water mol­ecules in a distorted octa­hedral geometry. The Co⋯Co separation is 5.1167 (7) Å. The coordinated and uncoordinated water mol­ecules engage in N—H⋯O and O—H⋯O hydrogen-bonding inter­actions.

## Related literature

For related compounds, see: Jing *et al.* (2000 ▶, 2004 ▶); Nagababu & Satyanarayana (2007 ▶); Selvi & Palaniandavar (2002 ▶); Shavaleev *et al.* (2007 ▶); Wang *et al.* (2008 ▶); Wu *et al.* (1997 ▶).
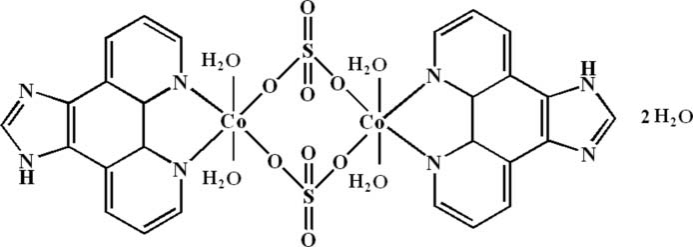


## Experimental

### 

#### Crystal data

[Co_2_(SO_4_)_2_(C_13_H_8_N_4_)_2_(H_2_O)_4_]·2H_2_O
*M* *_r_* = 858.54
Monoclinic, 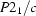

*a* = 10.3160 (13) Å
*b* = 9.0716 (10) Å
*c* = 16.8549 (17) Å
β = 99.1040 (10)°
*V* = 1557.5 (3) Å^3^
*Z* = 2
Mo *K*α radiation
μ = 1.29 mm^−1^
*T* = 298 K
0.43 × 0.36 × 0.22 mm

#### Data collection

Siemens SMART CCD area-detector diffractometer
Absorption correction: multi-scan (*SADABS*; Sheldrick, 1996 ▶) *T*_min_ = 0.581, *T*_max_ = 0.754
7558 measured reflections
2742 independent reflections
2329 reflections with *I* > 2σ(*I*)
*R*_int_ = 0.020

#### Refinement

*R*[*F* ^2^ > 2σ(*F* ^2^)] = 0.027
*wR*(*F* ^2^) = 0.073
*S* = 1.07
2742 reflections
235 parameters
H-atom parameters constrained
Δρ_max_ = 0.63 e Å^−3^
Δρ_min_ = −0.36 e Å^−3^

### 

Data collection: *SMART* (Siemens, 1996 ▶); cell refinement: *SAINT* (Siemens, 1996 ▶); data reduction: *SAINT*; program(s) used to solve structure: *SHELXS97* (Sheldrick, 2008 ▶); program(s) used to refine structure: *SHELXL97* (Sheldrick, 2008 ▶); molecular graphics: *SHELXTL* (Sheldrick, 2008 ▶); software used to prepare material for publication: *SHELXTL*.

## Supplementary Material

Crystal structure: contains datablocks global, I. DOI: 10.1107/S1600536809024295/ng2604sup1.cif

Structure factors: contains datablocks I. DOI: 10.1107/S1600536809024295/ng2604Isup2.hkl

Additional supplementary materials:  crystallographic information; 3D view; checkCIF report

## Figures and Tables

**Table 1:** Selected bond lengths (Å)

Co1—N1	2.124 (2)
Co1—N2	2.137 (2)
Co1—O6	2.0654 (19)
Co1—O1	2.0889 (17)
Co1—O2	2.0978 (17)
Co1—O5	2.1468 (18)

**Table 2:** Hydrogen-bond geometry (Å, °)

*D*—H⋯*A*	*D*—H	H⋯*A*	*D*⋯*A*	*D*—H⋯*A*
N3—H3⋯O4^i^	0.86	2.05	2.886 (3)	165
O5—H5*A*⋯O3	0.85	1.94	2.764 (2)	165
O6—H6*B*⋯O7^ii^	0.85	1.79	2.634 (3)	174
O7—H7*B*⋯O3^iii^	0.85	2.00	2.843 (3)	176

Symmetry codes: (i) 
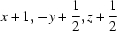
; (ii) 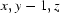; (iii) 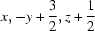.

**Fig. 1.**
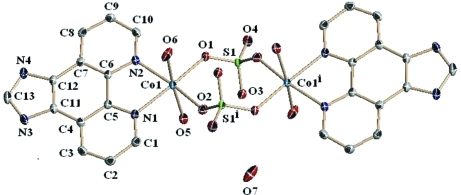
The structure of (I), with the atomic numbering scheme and displacement ellipsoids at the 30% probability level (H atoms omitted for clarity). [Symmetry codes: (i) -1-x,1-y,-z.]

**Fig. 2.**
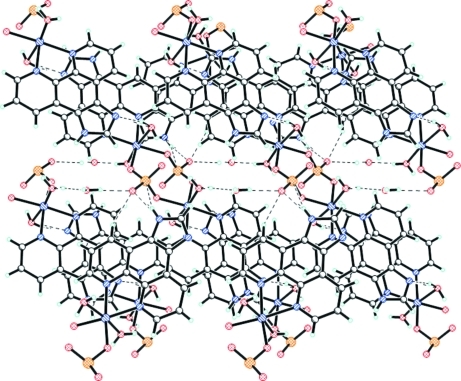
Three dimensional supramolecular architecture constructed by intermolecular hydrogen bonds. The dotted lines indicate the hydrogen bonds.

## supplementary crystallographic information

### Comment

Transitional metal complexes with the diimine ligands have potential applications in catalysis, molecular adsorption, magnetism, nonlinear optics, and molecular sensing. Imidazo[4,5-f]-1,10- phenanthroline (IP) ligand possesses good coordination ability due to the hetercyclic nitrogen atoms in the structure. The IP ligand is easy to prepare and modify and therefore have recently gained a lot of interest with respect to synthesis of its novel metal compounds. Most of its metal complexes are focused on Pt complexes (Shavaleev, *et al.*, 2007) and Ru complexes (Wu, *et al.*, 1997; Jing, *et al.*, 2000; Jing, *et al.*, 2004). Several Co-IP complexes have been reported in the presence of co-ligands such as 1,10-phenanthroline, 2,2'-bipyridine or ethylenediamine (Selvi, *et al.*, 2002; Nagababu, *et al.*, 2007). A Ni-IP complex has been reported recently (Wang, *et al.*, 2008), which exhibits a mononuclear structure. In the present paper, we hydrothermally synthesized a novel coordination complex constructed from CoSO_4_ and IP.

The molecular structure of the complex (I) (Fig. 1) has one Co(II), one IP, two coordinated water molecules, one sulfuric anion and one dissociated water molecule in its asymmetric unit. The Co(II) center is six-coordinated by two nitrogen atoms from two IP ligands, two oxygen atoms from water and two oxygen atoms from two sulfuric anions in a distorted octahedral geometry. Each IP ligand is chelate-coordinated to one Co(II) with two nitrogen atoms uncoordinated (Fig.1). Two sulfuric anions bridge two Co(II) centers to form a dinuclear cobalt clusters with the Co···Co separation of 5.1167 (7) Å (Fig. 1). Strong hydrogen bonds exist in the structure (Table 2).

Strong π–π stacking interactions are observed in the structure. For example: Aromatic phenyl rings 1 and 2 are composed of C4, C5, C6, C7, C11, C12 and C4A, C5A, C6A, C7A, C11A, C12A (atom with additional label A refers to the symmetry operation: 2- *x*, - *y*, - *z*). The perpendicular distance and the centroid-centroid distance are 3.41Å, and 4.251 (6)Å, respectively.

### Experimental

The ligand IP was synthesised according to the procedure published already (Wu, *et al.*, 1997). Yield: 87%. HNMR (DMSO-d6): 9.06(dd, 2H), 8.87(d, 2H), 8.50(s, 1H), 7.83(dd, 2H).

A mixture of IP (0.05 mmol, 0.011 g), CoSO_4_7H_2_O (0.1 mmol, 0.028 g), KSCN (0.1mmol, 0.010g ) and water (8 ml) was put into a Teflon-lined autoclave. The reaction mixture was heated at 120 centigrade for two day, followed by slow cooling to room temperatrue and brown single crystals were collected. Elemental analyse found: C, 36.36; H, 3.26; N, 13.05%.

### Refinement

H atoms were positioned geometrically and refined as riding atoms, with C—H = 0.93Å and *U*_iso_(H) = 1.2*U*_eq_(C) for methyl H atoms, N—H = 0.86Å and *U*_iso_(H) = 1.2*U*_eq_(C) for amino H atoms.

### Crystal data

**Table:**

[Co_2_(SO_4_)_2_(C_13_H_8_N_4_)_2_(H_2_O)_4_]·2H_2_O	*F*(000) = 876
*M**_r_* = 858.54	*D*_x_ = 1.831 Mg m^−^^3^
Monoclinic, *P*2_1_/*c*	Mo *K*α radiation, λ = 0.71073 Å
Hall symbol: -P 2ybc	Cell parameters from 7558 reflections
*a* = 10.3160 (13) Å	θ = 2.0–25.0°
*b* = 9.0716 (10) Å	µ = 1.29 mm^−^^1^
*c* = 16.8549 (17) Å	*T* = 298 K
β = 99.104 (1)°	Block, brown
*V* = 1557.5 (3) Å^3^	0.43 × 0.36 × 0.22 mm
*Z* = 2	

### Data collection

**Table:**

Siemens SMART CCD area-detector diffractometer	2742 independent reflections
Radiation source: fine-focus sealed tube	2329 reflections with *I* > 2σ(*I*)
graphite	*R*_int_ = 0.020
φ and ω scans	θ_max_ = 25.0°, θ_min_ = 2.0°
Absorption correction: multi-scan (*SADABS*; Sheldrick, 1996)	*h* = −12→8
*T*_min_ = 0.581, *T*_max_ = 0.754	*k* = −10→10
7558 measured reflections	*l* = −19→20

### Refinement

**Table:**

Refinement on *F*^2^	Primary atom site location: structure-invariant direct methods
Least-squares matrix: full	Secondary atom site location: difference Fourier map
*R*[*F*^2^ > 2σ(*F*^2^)] = 0.027	Hydrogen site location: inferred from neighbouring sites
*wR*(*F*^2^) = 0.073	H-atom parameters constrained
*S* = 1.07	*w* = 1/[σ^2^(*F*_o_^2^) + (0.0289*P*)^2^ + 1.5148*P*] where *P* = (*F*_o_^2^ + 2*F*_c_^2^)/3
2742 reflections	(Δ/σ)_max_ < 0.001
235 parameters	Δρ_max_ = 0.63 e Å^−^^3^
0 restraints	Δρ_min_ = −0.36 e Å^−^^3^

### Special details

**Table:**

Geometry. All esds (except the esd in the dihedral angle between two l.s. planes) are estimated using the full covariance matrix. The cell esds are taken into account individually in the estimation of esds in distances, angles and torsion angles; correlations between esds in cell parameters are only used when they are defined by crystal symmetry. An approximate (isotropic) treatment of cell esds is used for estimating esds involving l.s. planes.
Refinement. Refinement of F^2^ against ALL reflections. The weighted R-factor wR and goodness of fit S are based on F^2^, conventional R-factors R are based on F, with F set to zero for negative F^2^. The threshold expression of F^2^ > 2sigma(F^2^) is used only for calculating R-factors(gt) etc. and is not relevant to the choice of reflections for refinement. R-factors based on F^2^ are statistically about twice as large as those based on F, and R- factors based on ALL data will be even larger.

### Fractional atomic coordinates and isotropic or equivalent isotropic displacement parameters (Å^2^)

**Table:**

	*x*	*y*	*z*	*U*_iso_*/*U*_eq_	
Co1	0.68903 (3)	0.31926 (4)	0.04124 (2)	0.02494 (12)	
N1	0.8745 (2)	0.3237 (2)	0.11677 (12)	0.0266 (5)	
N2	0.75649 (19)	0.0980 (2)	0.03284 (12)	0.0254 (5)	
N3	1.2464 (2)	−0.0127 (2)	0.20274 (13)	0.0326 (5)	
H3	1.3069	0.0380	0.2315	0.039*	
N4	1.1436 (2)	−0.2056 (2)	0.13930 (13)	0.0339 (5)	
O1	0.53904 (16)	0.29041 (18)	−0.05637 (10)	0.0302 (4)	
O2	0.63548 (17)	0.53058 (18)	0.07489 (11)	0.0339 (4)	
O3	0.56078 (16)	0.51259 (19)	−0.13340 (10)	0.0307 (4)	
O4	0.40919 (19)	0.3224 (2)	−0.18590 (11)	0.0399 (5)	
O5	0.79510 (17)	0.4343 (2)	−0.03947 (10)	0.0323 (4)	
H5A	0.7307	0.4726	−0.0703	0.039*	
H5B	0.8276	0.3690	−0.0667	0.039*	
O6	0.5767 (2)	0.2368 (2)	0.12221 (12)	0.0472 (5)	
H6A	0.5151	0.2917	0.1330	0.057*	
H6B	0.5745	0.1555	0.1470	0.057*	
O7	0.5866 (3)	0.9854 (2)	0.20115 (13)	0.0658 (7)	
H7A	0.5836	0.8952	0.1873	0.079*	
H7B	0.5822	0.9896	0.2510	0.079*	
S1	0.46794 (6)	0.39915 (6)	−0.11220 (4)	0.02442 (15)	
C1	0.9322 (3)	0.4409 (3)	0.15383 (16)	0.0312 (6)	
H1	0.8867	0.5298	0.1501	0.037*	
C2	1.0586 (3)	0.4365 (3)	0.19832 (16)	0.0335 (6)	
H2	1.0963	0.5212	0.2232	0.040*	
C3	1.1263 (3)	0.3062 (3)	0.20491 (16)	0.0322 (6)	
H3A	1.2097	0.3008	0.2352	0.039*	
C4	1.0685 (2)	0.1811 (3)	0.16548 (14)	0.0255 (5)	
C5	0.9415 (2)	0.1946 (3)	0.12104 (14)	0.0239 (5)	
C6	0.8762 (2)	0.0708 (3)	0.07713 (14)	0.0231 (5)	
C7	0.9348 (2)	−0.0701 (3)	0.08041 (14)	0.0247 (5)	
C8	0.8635 (3)	−0.1852 (3)	0.03828 (16)	0.0304 (6)	
H8	0.8987	−0.2797	0.0390	0.036*	
C9	0.7410 (3)	−0.1567 (3)	−0.00397 (16)	0.0333 (6)	
H9	0.6916	−0.2323	−0.0310	0.040*	
C10	0.6914 (2)	−0.0137 (3)	−0.00605 (15)	0.0305 (6)	
H10	0.6092	0.0044	−0.0359	0.037*	
C11	1.1259 (2)	0.0376 (3)	0.16564 (15)	0.0265 (5)	
C12	1.0637 (2)	−0.0818 (3)	0.12648 (15)	0.0260 (5)	
C13	1.2505 (3)	−0.1568 (3)	0.18502 (17)	0.0378 (7)	
H13	1.3222	−0.2167	0.2033	0.045*	

### Atomic displacement parameters (Å^2^)

**Table:**

	*U*^11^	*U*^22^	*U*^33^	*U*^12^	*U*^13^	*U*^23^
Co1	0.02401 (19)	0.02377 (19)	0.0260 (2)	0.00539 (14)	0.00088 (13)	0.00188 (14)
N1	0.0291 (11)	0.0243 (11)	0.0257 (11)	0.0054 (9)	0.0026 (9)	0.0006 (9)
N2	0.0233 (11)	0.0275 (11)	0.0246 (11)	0.0037 (9)	0.0015 (8)	0.0013 (9)
N3	0.0276 (11)	0.0378 (12)	0.0288 (12)	0.0042 (10)	−0.0068 (9)	−0.0011 (10)
N4	0.0355 (12)	0.0316 (12)	0.0328 (13)	0.0118 (10)	0.0004 (10)	0.0020 (10)
O1	0.0297 (9)	0.0255 (9)	0.0323 (10)	0.0025 (7)	−0.0046 (8)	0.0043 (8)
O2	0.0317 (10)	0.0241 (9)	0.0478 (11)	0.0049 (8)	0.0117 (8)	−0.0021 (8)
O3	0.0310 (9)	0.0289 (9)	0.0325 (10)	0.0035 (8)	0.0061 (8)	0.0049 (8)
O4	0.0504 (12)	0.0338 (10)	0.0302 (10)	0.0044 (9)	−0.0102 (9)	−0.0075 (8)
O5	0.0311 (10)	0.0350 (10)	0.0307 (10)	0.0052 (8)	0.0044 (8)	0.0005 (8)
O6	0.0587 (13)	0.0317 (10)	0.0586 (14)	0.0165 (10)	0.0318 (11)	0.0167 (10)
O7	0.129 (2)	0.0306 (11)	0.0426 (13)	0.0143 (13)	0.0287 (14)	0.0071 (10)
S1	0.0258 (3)	0.0217 (3)	0.0239 (3)	0.0051 (2)	−0.0016 (2)	−0.0010 (2)
C1	0.0388 (15)	0.0237 (13)	0.0308 (14)	0.0034 (11)	0.0046 (11)	−0.0012 (11)
C2	0.0372 (15)	0.0291 (14)	0.0335 (15)	−0.0045 (12)	0.0029 (12)	−0.0052 (11)
C3	0.0273 (13)	0.0356 (15)	0.0315 (15)	−0.0006 (11)	−0.0023 (11)	−0.0032 (12)
C4	0.0254 (13)	0.0284 (13)	0.0224 (13)	0.0026 (11)	0.0025 (10)	0.0003 (10)
C5	0.0257 (13)	0.0232 (12)	0.0228 (13)	0.0039 (10)	0.0037 (10)	0.0014 (10)
C6	0.0246 (12)	0.0233 (12)	0.0211 (12)	0.0029 (10)	0.0029 (10)	0.0020 (10)
C7	0.0275 (13)	0.0249 (12)	0.0219 (13)	0.0021 (10)	0.0046 (10)	0.0035 (10)
C8	0.0370 (14)	0.0219 (13)	0.0320 (14)	0.0029 (11)	0.0051 (11)	0.0001 (11)
C9	0.0349 (15)	0.0283 (14)	0.0349 (15)	−0.0056 (11)	0.0002 (12)	−0.0039 (12)
C10	0.0245 (13)	0.0341 (14)	0.0309 (14)	0.0003 (11)	−0.0015 (10)	−0.0023 (12)
C11	0.0245 (12)	0.0303 (13)	0.0240 (13)	0.0058 (11)	0.0014 (10)	0.0030 (11)
C12	0.0284 (13)	0.0267 (13)	0.0230 (13)	0.0071 (10)	0.0047 (10)	0.0029 (10)
C13	0.0368 (16)	0.0401 (16)	0.0341 (16)	0.0169 (13)	−0.0021 (12)	0.0036 (12)

### Geometric parameters (Å, °)

**Table:**

Co1—N1	2.124 (2)	O7—H7A	0.8500
Co1—N2	2.137 (2)	O7—H7B	0.8500
Co1—O6	2.0654 (19)	S1—O2^i^	1.4671 (18)
Co1—O1	2.0889 (17)	C1—C2	1.398 (4)
Co1—O2	2.0978 (17)	C1—H1	0.9300
Co1—O5	2.1468 (18)	C2—C3	1.369 (4)
N1—C1	1.326 (3)	C2—H2	0.9300
N1—C5	1.356 (3)	C3—C4	1.400 (3)
N2—C10	1.330 (3)	C3—H3A	0.9300
N2—C6	1.360 (3)	C4—C5	1.408 (3)
N3—C13	1.343 (3)	C4—C11	1.430 (3)
N3—C11	1.378 (3)	C5—C6	1.451 (3)
N3—H3	0.8600	C6—C7	1.411 (3)
N4—C13	1.318 (4)	C7—C8	1.404 (3)
N4—C12	1.390 (3)	C7—C12	1.433 (3)
O1—S1	1.4758 (17)	C8—C9	1.373 (4)
O2—S1^i^	1.4671 (18)	C8—H8	0.9300
O3—S1	1.4873 (18)	C9—C10	1.393 (4)
O4—S1	1.4689 (18)	C9—H9	0.9300
O5—H5A	0.8500	C10—H10	0.9300
O5—H5B	0.8501	C11—C12	1.374 (3)
O6—H6A	0.8499	C13—H13	0.9300
O6—H6B	0.8499		
			
O6—Co1—O1	92.97 (8)	N1—C1—C2	122.7 (2)
O6—Co1—O2	87.31 (7)	N1—C1—H1	118.6
O1—Co1—O2	97.67 (7)	C2—C1—H1	118.6
O6—Co1—N1	99.04 (8)	C3—C2—C1	119.3 (2)
O1—Co1—N1	163.56 (8)	C3—C2—H2	120.4
O2—Co1—N1	94.09 (7)	C1—C2—H2	120.4
O6—Co1—N2	85.76 (8)	C2—C3—C4	119.2 (2)
O1—Co1—N2	92.21 (7)	C2—C3—H3A	120.4
O2—Co1—N2	168.21 (8)	C4—C3—H3A	120.4
N1—Co1—N2	77.62 (7)	C3—C4—C5	118.2 (2)
O6—Co1—O5	172.06 (7)	C3—C4—C11	126.3 (2)
O1—Co1—O5	87.15 (7)	C5—C4—C11	115.5 (2)
O2—Co1—O5	84.80 (7)	N1—C5—C4	121.8 (2)
N1—Co1—O5	82.50 (7)	N1—C5—C6	116.8 (2)
N2—Co1—O5	102.17 (7)	C4—C5—C6	121.4 (2)
C1—N1—C5	118.7 (2)	N2—C6—C7	122.1 (2)
C1—N1—Co1	126.32 (17)	N2—C6—C5	116.6 (2)
C5—N1—Co1	114.74 (16)	C7—C6—C5	121.3 (2)
C10—N2—C6	118.4 (2)	C8—C7—C6	117.8 (2)
C10—N2—Co1	127.18 (16)	C8—C7—C12	125.9 (2)
C6—N2—Co1	114.27 (16)	C6—C7—C12	116.3 (2)
C13—N3—C11	106.2 (2)	C9—C8—C7	119.2 (2)
C13—N3—H3	126.9	C9—C8—H8	120.4
C11—N3—H3	126.9	C7—C8—H8	120.4
C13—N4—C12	103.9 (2)	C8—C9—C10	119.6 (2)
S1—O1—Co1	130.53 (10)	C8—C9—H9	120.2
S1^i^—O2—Co1	138.89 (11)	C10—C9—H9	120.2
Co1—O5—H5A	99.1	N2—C10—C9	122.8 (2)
Co1—O5—H5B	106.7	N2—C10—H10	118.6
H5A—O5—H5B	107.0	C9—C10—H10	118.6
Co1—O6—H6A	116.5	C12—C11—N3	106.0 (2)
Co1—O6—H6B	134.5	C12—C11—C4	123.7 (2)
H6A—O6—H6B	108.9	N3—C11—C4	130.2 (2)
H7A—O7—H7B	108.0	C11—C12—N4	110.0 (2)
O2^i^—S1—O4	109.74 (11)	C11—C12—C7	121.7 (2)
O2^i^—S1—O1	109.81 (11)	N4—C12—C7	128.4 (2)
O4—S1—O1	108.62 (10)	N4—C13—N3	113.8 (2)
O2^i^—S1—O3	109.99 (10)	N4—C13—H13	123.1
O4—S1—O3	108.67 (11)	N3—C13—H13	123.1
O1—S1—O3	109.98 (10)		
			
O6—Co1—N1—C1	−100.4 (2)	Co1—N1—C5—C6	−2.7 (3)
O1—Co1—N1—C1	123.2 (3)	C3—C4—C5—N1	−1.0 (4)
O2—Co1—N1—C1	−12.5 (2)	C11—C4—C5—N1	178.9 (2)
N2—Co1—N1—C1	176.0 (2)	C3—C4—C5—C6	178.7 (2)
O5—Co1—N1—C1	71.7 (2)	C11—C4—C5—C6	−1.5 (4)
O6—Co1—N1—C5	84.93 (18)	C10—N2—C6—C7	2.5 (4)
O1—Co1—N1—C5	−51.5 (3)	Co1—N2—C6—C7	178.45 (18)
O2—Co1—N1—C5	172.86 (17)	C10—N2—C6—C5	−177.7 (2)
N2—Co1—N1—C5	1.34 (17)	Co1—N2—C6—C5	−1.7 (3)
O5—Co1—N1—C5	−102.94 (18)	N1—C5—C6—N2	3.0 (3)
O6—Co1—N2—C10	75.6 (2)	C4—C5—C6—N2	−176.7 (2)
O1—Co1—N2—C10	−17.2 (2)	N1—C5—C6—C7	−177.2 (2)
O2—Co1—N2—C10	129.8 (3)	C4—C5—C6—C7	3.1 (4)
N1—Co1—N2—C10	175.8 (2)	N2—C6—C7—C8	−2.2 (4)
O5—Co1—N2—C10	−104.8 (2)	C5—C6—C7—C8	178.0 (2)
O6—Co1—N2—C6	−99.96 (17)	N2—C6—C7—C12	177.3 (2)
O1—Co1—N2—C6	167.22 (17)	C5—C6—C7—C12	−2.5 (3)
O2—Co1—N2—C6	−45.8 (4)	C6—C7—C8—C9	0.0 (4)
N1—Co1—N2—C6	0.26 (16)	C12—C7—C8—C9	−179.4 (3)
O5—Co1—N2—C6	79.66 (17)	C7—C8—C9—C10	1.7 (4)
O6—Co1—O1—S1	117.94 (15)	C6—N2—C10—C9	−0.6 (4)
O2—Co1—O1—S1	30.25 (15)	Co1—N2—C10—C9	−176.0 (2)
N1—Co1—O1—S1	−105.0 (3)	C8—C9—C10—N2	−1.5 (4)
N2—Co1—O1—S1	−156.20 (15)	C13—N3—C11—C12	−0.4 (3)
O5—Co1—O1—S1	−54.11 (15)	C13—N3—C11—C4	178.7 (3)
O6—Co1—O2—S1^i^	−21.15 (18)	C3—C4—C11—C12	179.2 (3)
O1—Co1—O2—S1^i^	71.49 (18)	C5—C4—C11—C12	−0.7 (4)
N1—Co1—O2—S1^i^	−120.03 (18)	C3—C4—C11—N3	0.2 (5)
N2—Co1—O2—S1^i^	−75.2 (4)	C5—C4—C11—N3	−179.7 (2)
O5—Co1—O2—S1^i^	157.89 (18)	N3—C11—C12—N4	0.4 (3)
Co1—O1—S1—O2^i^	−80.84 (16)	C4—C11—C12—N4	−178.8 (2)
Co1—O1—S1—O4	159.15 (14)	N3—C11—C12—C7	−179.6 (2)
Co1—O1—S1—O3	40.34 (17)	C4—C11—C12—C7	1.2 (4)
C5—N1—C1—C2	−1.1 (4)	C13—N4—C12—C11	−0.2 (3)
Co1—N1—C1—C2	−175.59 (19)	C13—N4—C12—C7	179.7 (3)
N1—C1—C2—C3	−0.5 (4)	C8—C7—C12—C11	179.9 (2)
C1—C2—C3—C4	1.4 (4)	C6—C7—C12—C11	0.4 (4)
C2—C3—C4—C5	−0.7 (4)	C8—C7—C12—N4	−0.1 (4)
C2—C3—C4—C11	179.5 (3)	C6—C7—C12—N4	−179.5 (2)
C1—N1—C5—C4	1.8 (4)	C12—N4—C13—N3	0.0 (3)
Co1—N1—C5—C4	176.97 (18)	C11—N3—C13—N4	0.3 (3)
C1—N1—C5—C6	−177.8 (2)		

Symmetry codes: (i) −*x*+1, −*y*+1, −*z*.

### Hydrogen-bond geometry (Å, °)

**Table:**

*D*—H···*A*	*D*—H	H···*A*	*D*···*A*	*D*—H···*A*
N3—H3···O4^ii^	0.86	2.05	2.886 (3)	165
N3—H3···S1^ii^	0.86	2.94	3.709 (2)	150
O5—H5A···O3	0.85	1.94	2.764 (2)	165
O5—H5A···S1	0.85	2.77	3.4184 (19)	134
O6—H6B···O7^iii^	0.85	1.79	2.634 (3)	174
O7—H7B···O3^iv^	0.85	2.00	2.843 (3)	176
O7—H7B···S1^iv^	0.85	2.93	3.703 (3)	152

Symmetry codes: (ii) *x*+1, −*y*+1/2, *z*+1/2;  (iii) *x*, *y*−1, *z*; (iv) *x*, −*y*+3/2, *z*+1/2.
